# Reduced Cortisol in Boys with Early-Onset Conduct Disorder and Callous-Unemotional Traits

**DOI:** 10.1155/2013/349530

**Published:** 2013-06-11

**Authors:** Georg G. von Polier, Beate Herpertz-Dahlmann, Kerstin Konrad, Kristine Wiesler, Jana Rieke, Monika Heinzel-Gutenbrunner, Christian J. Bachmann, Timo D. Vloet

**Affiliations:** ^1^Department of Child and Adolescent Psychiatry, Medical Faculty, RWTH Aachen University, Neuenhofer Weg 21, 52074 Aachen, Germany; ^2^Child Neuropsychology Section, Department of Child and Adolescent Psychiatry, Medical Faculty, RWTH Aachen University, Pauwelsstraße 30, 52074 Aachen, Germany; ^3^JARA Translational Medicine, Aachen and Jülich, 52425 Jülich, Germany; ^4^Department of Child and Adolescent Psychiatry, Universitätsklinikum Gießen und Marburg GmbH, Campus Marburg, Hans-Sachs-Straße 4, 35039 Marburg, Germany; ^5^Department of Child and Adolescent Psychiatry, Charité-Universitätsmedizin Berlin, Charitéplatz 1, 10117 Berlin, Germany

## Abstract

*Background*. A growing body of evidence suggests an association between altered hypothalamic-pituitary-adrenal axis reactivity and the development of persistent antisocial behavior in children. However the effects of altered cortisol levels remain poorly understood in the complex context of conduct disorder, callous-unemotional (CU) personality traits, and frequent comorbidities, such as attention deficit hyperactivity disorder (ADHD). The aim of the current study was to investigate associations among CU traits, antisocial behavior, and comorbid ADHD symptomatology with cortisol levels in male children and adolescents. *Methods*. The study included 37 boys with early-onset conduct disorder (EO-CD, mean age 11.9 years) and 38 healthy boys (mean age 12.5 years). Participants were subjected to multiple daytime salivary cortisol measurements and a psychometric characterization. *Results*. Subjects in the EO-CD group with elevated CU traits showed a diminished cortisol awakening response compared to healthy participants. In the EO-CD group, high CU traits and impulsivity were associated with decreased diurnal cortisol levels, while associations with antisocial behavior were not detected. The cortisol awakening response was significantly inversely associated with hyperactivity (*P* = 0.02) and marginally significant with CU traits (*P* = 0.07). *Conclusions*. These results indicate a specific association between CU traits and a diminished stress response, which is not explained by antisocial behavior in general.

## 1. Introduction

In recent years, multiple studies have indicated that children with persistent antisocial behavior show neurobiological alterations [1]. In particular, stress-regulating mechanisms appear to play a major role in the development of antisocial behavior [2]. Many studies in this area have focused on the hypothalamic-pituitary-adrenal (HPA) axis as a central component of the stress-regulating system and, in particular, on cortisol as the primary stress hormone in humans.

Evidence from nonhuman animals indicates that abolishing the hormonal response to stress may impair processing of social signals and lead to abnormal patterns of aggression [3]. One theory focuses on stress thresholds and sensation-seeking behavior [4] and argues that antisocial individuals have elevated thresholds for stress. They are said to be more easily bored and might abate this state of low arousal through acting out antisocial behavior. 

However, experimental findings regarding the relationship between antisocial behavior and cortisol are contradictory. Some researchers found antisocial behavior to be associated with diminished basal cortisol levels [5], while other studies failed to identify any relationship [6] or report increased basal cortisol levels [7]. A meta-analysis [8] found reduced basal cortisol levels to be associated with antisocial behavior at small effect sizes, and primarily these associations were detected in clinical populations of school-aged children. Conflicting findings of the aforementioned studies may be attributed to methodological variations, for example, assessment methods (single measurement versus multiple daily measurements; plasma versus saliva samples), the use of diverse informants (self-report versus parental or teacher report), or sample sizes. A particular problem in comparing the neuroendocrinological data of antisocial children and adolescents is the heterogeneity of psychiatric diagnoses in the investigated cohorts, which range from healthy children [9] to children with disruptive behavior disorder (DBD) [10] or children with conduct disorder (CD) [7]. Moreover, children and adolescents with DBD or CD are frequently diagnosed with comorbid attention deficit hyperactivity disorder (ADHD) [11]. In particular, hyperactive and impulsive symptoms have been found to be associated with reduced diurnal cortisol levels [12, 13], though not consistently [14].

More consistent findings on a possible association of cortisol levels and antisocial behavior have been yielded in studies investigating the relationship of cortisol and psychopathic traits in adolescent [15] and adult cohorts with severe antisocial behavior [16]. Psychopathy is defined as a personality disorder that amongst others is marked by emotional deficits such as reduced feelings of guilt and lack of empathy, also referred to as callous and unemotional (CU) traits. CU traits designate a group of youth with a particularly severe, aggressive, and stable pattern of antisocial behavior [17] and have been proposed as a specifier within the diagnostic category of CD in the DSM-5. Cortisol is believed to play an important role in the development and the stability of CU traits in that it maintains strong connections with limbic structures affecting brain activation patterns by changing the excitability of cell membranes [18, 19]. Apart from the hypothalamus, particularly the amygdala is highly involved in the HPA axis activity [20]. A reduced activation of the amygdala has been associated with diminished emotional learning in general and specifically in subjects with CU traits [21]. Other traits characteristic for individuals with CU traits such as reduced empathy and a lower punishment sensitivity have been associated with reduced cortisol levels as well [22].

Studies on cortisol in adults with psychopathic traits point toward an inversely proportional association between psychopathic traits and both daily cortisol levels [16] and stress-induced cortisol reactivity [23, 24]. However, other investigations have failed to detect a relationship between cortisol levels and psychopathic traits in adults [25, 26]. In children and adolescents, a few studies have reported similar results. Morning cortisol levels [15] and stress-induced cortisol reactivity [27] have been found to be reduced in subjects with elevated CU traits. One study did not find an association between cortisol and CU traits [28] but did indicate a negative association between cortisol levels and psychopathy related impulsivity in boys. The latter study investigated a community sample of 15-year-old adolescents; however plasma cortisol was assessed one time only in the afternoon and awakening time was not controlled for. In previous studies analyzing the relationship between CU traits and cortisol levels, a possible impact of externalizing behavior [8] and ADHD related pathology has not been considered and might vary depending on the subjects studied. Moreover, the cortisol awakening response (CAR) that shows strong associations with limbic functioning [29] has not been investigated with regard to CU traits to date. 

Thus, the aim of this study was to analyze associations of the CAR and the diurnal rhythm of cortisol secretion with CU traits. Moreover, it was attempted to disentangle a possible impact of externalizing behavior and ADHD related pathology. In order to examine participants with high levels of antisocial behavior and elevated rates of comorbid ADHD, this study was conducted in a clinical setting in children and adolescents with early-onset conduct disorder (EO-CD, onset before the age of 10 years [30]). Children diagnosed with EO-CD have a particularly poor prognosis that might be related to neurobiological factors [31, 32]. Based on the aforementioned findings, the following were assumed: first, compared to healthy controls, boys with EO-CD show a reduced HPA axis activity. Second, in boys with EO-CD, CU traits are associated with reduced cortisol levels. In addition, the influence of ADHD will be explored.

## 2. Methods

### 2.1. Participants

This study involved a total of 75 boys aged 7 to 16 years (mean age 12.2; SD = 2.5). Of these, 37 subjects fulfilled the diagnostic criteria for EO-CD (according to the DSM-IV-TR [30]), and 38 boys constituted a healthy control (HC) group. Subjects with EO-CD were consecutively included at a university hospital (which is obliged to treat any patient from a defined region of 850. 000 inhabitants) from all inpatient and outpatient referrals with suspected CD. Subjects in the healthy control group were recruited by announcements in local schools without disclosing the aim of the study. All participants and their legally appointed guardians gave written informed consent, and the study was approved by the local ethics committee in accordance with the Declaration of Helsinki. Subjects were excluded from the study if they had a general IQ below 80 (WISC-IV, German version by [33]), evidence of a neurological disorder, or a history or current diagnosis of psychosis, trauma, bipolar disorder, substance abuse, or pervasive developmental disorder. Further exclusion criteria were any chronic physical illness and the use of any medication with the exception of methylphenidate.

All participants were assessed for CD, oppositional defiant disorder (ODD), ADHD, major depressive disorder (MDD), anxiety disorders (GAD/SAD), obsessive-compulsive disorder (OCD), tic disorder, elimination disorder, and posttraumatic stress disorder (PTSD) using the Schedule for Affective Disorders and Schizophrenia for School-Age Children-Present and Lifetime Version (K-SADS-PL, [34, 35]), which reflects the DSM-IV criteria. Senior child and adolescent psychiatrists performed the diagnostic interviews with the main caregivers of all participants and the participants themselves. Participants with suspected CD symptomatology were included if caregivers reported at least three CD symptoms (out of 15 different antisocial behaviors) with at least one symptom beginning before age 10 and if functional impairment was present [30]. Comorbidity in the EO-CD group was as follows: 25 participants (67%) were diagnosed with ADHD (24 of the combined subtype, 1 of the inattentive subtype, according to the DSM-IV-TR); 1 participant with MDD; 1 participant with SAD; 1 participant with tic disorder; 5 participants (14%) with an elimination disorder, all according to the DSM-IV-TR [30]. All participants in the healthy control did not fulfill the criteria for any psychiatric disorder. 

A total of 13 of the subjects with EO-CD received methylphenidate treatment upon onset of the investigation. Medication with methylphenidate was discontinued 48 h before the cortisol assessment, even though methylphenidate treatment has previously been shown to have no effect on cortisol levels [36]. The influence of other factors that may modulate cortisol levels, such as age, pubertal stage, body mass index (BMI), adverse life events [37], and internalizing behavior, was also evaluated. 

### 2.2. Instruments

Psychopathic traits were measured by the Antisocial Personality Screening Device (APSD, [38]), a 20-item rating scale that assesses CU traits, narcissism, and impulsivity. In this study, parents served as informant and the CU traits subscale alone was evaluated. *T* values are presented according to the norms provided in the manual [38]. To assess the severity of ADHD symptoms, parents completed the German Parental and Teacher Report on ADHD symptoms (FBB-ADHS), which is part of the Diagnostic System of Mental Disorders in Children and Adolescents (DISYPS-II) [39] and has a favorable internal consistency and retest reliability [40]. The FBB-ADHS includes all of the items that are given in the diagnostic categories of the ADHD subtypes described by the DSM-IV. To assess the extent of ADHD symptomatology in the subscales of inattention, hyperactivity, and impulsivity, the sum scores of corresponding items in the clinical range were calculated. Adverse events have been linked to cortisol measures, especially cortisol reactivity [41], and were thus controlled for using the Life Events Checklist [42] that is part of the Clinician Administered PTSD Scale (CAPS) [43, 44]. All participants completed the Life Events Checklist with support from research staff when needed. The Child Behavior Checklist (CBCL; [45, 46]) was completed by parents of all participants. In this study, only the externalizing problem behavior scales (delinquency, aggression) and the internalizing problem scale were utilized, and German norms [46] were used to calculate *T* values. The stage of pubertal maturation was assessed by the self-ratings of participants using standardized figure drawings depicting Tanner's sexual maturation scale (score range: 1–5).

### 2.3. Cortisol Collection and Analyses

In all subjects, saliva for cortisol assessment was sampled following the protocol described by Popma and coworkers [10]. In detail, saliva was collected immediately after awakening (probe 1), 30 and 60 min after awakening (probes 2 and 3), at 12:00 (before lunch; probe 4), at 15:20 and 15:40 (mean = probe 5), and at 19:30 (approximately 12 h after mean awakening, probe 6) using the Salivette sampling device (Sarstedt, Nümbrecht, Germany). The collection procedure in inpatients was performed by a study nurse, thus ensuring a high adherence to the protocol. Saliva collection in CD outpatients and in the healthy control group was conducted on a weekday. All subjects spent the morning and early afternoon at school, including the inpatients at the hospital school. Participants and their parents were instructed both orally and in writing about the collection procedure. A study nurse recapitulated the procedure the day before assessment and gave personal assistance to collect the saliva. Study nurses completed a journal reporting the collection times of the saliva probes and were asked to report specific stressful events. 

Before taking the saliva probes, participants rinsed their mouths with water and then waited 1 min before producing each probe. They were asked to avoid smoking, eating, drinking caffeinated or alcoholic drinks, engaging in vigorous exercise, or brushing their teeth until the first three probes had been collected. Compliance was monitored using the journal. All probes were frozen immediately after collection and stored at −60°C until they were assayed in the laboratory. Cortisol was measured by enzyme-linked immunosorbent assays (Active Cortisol EIA, Diagnostic Systems Laboratories, Sinsheim, Germany) of 20-*μ*L duplicates of unextracted saliva probes. The intra-assay and interassay coefficients of variation were 4.78% and 7.35%, respectively. The results are reported in nmol/L.

### 2.4. Statistical Analyses

Independent *t*-tests were used to assess group differences in demographic variables and symptom scores. Analyses of the HPA axis activity were conducted using two summary measures: (i) the area under the curve day measurement (AUC_*d*_) of cortisol levels during the day, including probe 1 (awakening), probe 4 (12:00), probe 5 (mean of 15:20 and 15:40 probes), and probe 6 (19:30) and (ii) the area under the curve awakening measurement (AUC_*a*_) including probe 1 (awakening), probe 2 (+30 min), and probe 3 (+60 min). All AUC measures were calculated with reference to zero. The formula for the calculation of the AUC was derived from the trapezoid formula [47]. In a number of participants (*N* = 23), particularly in younger participants, the first salivary probe was revealed to contain the highest cortisol value, which has been reported in other studies with larger samples [48]. In these cases, the calculation of the AUC_*i*_ (increase) with reference to the cortisol level at awakening would have resulted in negative values. As negative values of the AUC_*i*_ would have distorted the analyses, the evaluation of the AUC_*i*_ was omitted. To control for possible confounding influences of relevant variables on cortisol measures, Pearson correlations were calculated between AUC_*d*_ and AUC_*a*_, respectively, and BMI, internalizing behavior, stage of pubertal development, life events, and age. To compare cortisol measures between the HC group and the EO-CD group, independent *t*-tests and MANCOVA analyses were calculated. The MANCOVA analysis included both the AUC_*d*_ and AUC_*a*_ as dependent variables and possible influence factors as covariates (i.e., BMI, internalizing behavior, stage of pubertal development, life events, and age). 

Changes of (log)-cortisol levels during the course of the day were assessed by repeated measures ANOVAs (RMANOVA) with “time” as a within subjects factor (same probes used as in the calculation of AUC_*d*_) and “group” (e.g., EO-CD versus HC) as a between subjects factor. Raw cortisol values were positively skewed and normalized using log_e_-transformation (after adding 1 to avoid the log of zero). When the assumption of sphericity was violated, degrees of freedom were corrected using the Greenhouse-Geisser procedure [49]. The main effects of “time” and “group” as well as the interactions between “time” and group' were analyzed using difference contrast tests. Effect sizes were reported as partial eta squared values (*η*_*P*_^2^). Further RMANCOVA was run including IQ, externalizing behavior, CU traits, hyperactivity, and impulsivity both a full model and in separate models to assess a possible impact of these variables on diurnal cortisol variation. 

In the EO-CD group, multiple linear regressions were calculated with AUC_*d*_ and AUC_*a*_, respectively, as a dependent variable. In order to evaluate all predictors of interest (i.e., externalizing behavior, CU-traits, hyperactivity, and impulsivity), backward variable selection was performed by sequentially dropping nonrelevant predictors, if any. A *P* value <0.10 was taken as the criterion to include an independent variable as a possible predictor. 

Data from subjects with awakening times above or below two SDs of the mean were excluded, which was applicable to two subjects from the HC group. Data from one subject in the EO-CD-group could not be analyzed due to technical problems in the laboratory cortisol analyses. The final sample thus consisted of 36 participants in the EO-CD-group and 36 participants in the HC group.

## 3. Results

### 3.1. Sample Description

Both groups were comparable with respect to age, pubertal status, and awakening time (Table 1). The EO-CD group had an average bedtime of 21:20; children in the HC group had an average bedtime of 21:55. Similar to the awakening time, average bedtimes were not different at a statistical level. Externalizing behavior, internalizing behavior, hyperactivity, impulsivity, and CU traits were more pronounced in the EO-CD group, whereas IQ was higher in the HC-group (Table 1). Two-sided Pearson correlations between relevant influence factors (i.e., age, pubertal status, internalizing behavior, BMI, IQ, and adverse life events) and AUC_*a*_ and AUC_*d*_ in the complete study sample and in the EO-CD group only revealed no statistically significant associations with cortisol levels (all *P* values > 0.1).

### 3.2. Cortisol Profiles

#### 3.2.1. Group Differences and Antisocial Behavior

**Figure 1:**
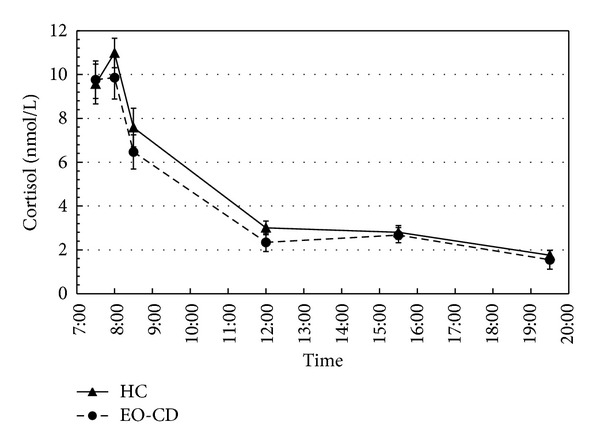
Group comparison of cortisol levels at awakening and throughout the day. Awakening and diurnal cortisol levels of subjects in the EO-CD group and the HC group are depicted. Significantly lower cortisol levels in the EO-CD group are detected at 12:00 only. Saliva cortisal measures were performed at awakening, +30 min, +60 min, at 12:00, 15:20, 15:40 (mean value of 15:20 and 15:40 depicted), and at 19:30. HC group = healthy control group; EO-CD = early-onset conduct disorder group; means and standard error bars are depicted.

A graphic representation of diurnal cortisol levels in both groups is given in Figure 1. Both diurnal cortisol levels (AUC_*d*_) and cortisol levels in the first hour after awakening (AUC_*a*_) did not differ significantly between the EO-CD and the HC groups (Table 1). AUC_*d*_ and AUC_*a*_ correlated with each other (*r* = 0.78; *P* < 0.001) and were entered as depending variables in a MANCOVA analysis with group (EO-CD/HC-group) as a fixed factor and relevant influence factors as covariates (i.e., age, pubertal status, internalizing behavior, BMI, IQ, and adverse life events). In both, a full model including all influence factors and separate models with one influence factor each, no significant group differences occurred. Also, no significant associations were detected with any possible influence factor.

A RMANOVA for the diurnal cycle (probes 1, 4, 5, and 7, excluding the CAR) revealed a significant main effect of time (*F* = 207.5, *P* < 0.001, *η*_*P*_^2^ = 0.75), a significant “time” × “group” interaction (*F* = 3.0, *P* = 0.03, *η*_*P*_^2^ = 0.04), but no main effect of group (*F* = 2.2, *P* = 0.14). Difference contrast tests showed significant decreases in log (cortisol) for each probe compared with the previous probes (all *P* < 0.001). The significant group by time interaction was mainly attributable to a significant difference between groups in the decline between probes one and four (*F* = 8.0; *P* = 0.006). When comparing log (cortisol) levels between groups at individual time points, a significant difference occurred at noon only (*T* = 2.5; *P* = 0.02). 

A MANOVA comparing awakening cortisol levels (AUC_*a*_) and diurnal cortisol levels (AUC_*d*_) between subjects in the EO-CD group with elevated CU traits (CU+ group; T value ≥ 70) and subjects of the HC group revealed marginally significant reduced cortisol levels in the CU+ group in multivariate testing (*P* = 0.076). In subsequent ANOVAs, awakening cortisol levels were significantly reduced in the CU+ group (*P* = 0.023; mean AUC_*a*_: 496 nmol/L∗h, SD = 151 nmol/L∗h), while diurnal cortisol levels were not significantly reduced in the CU+ group (*P* = 0.13; mean AUC_*d*_ = 2376 nmol/h; SD = 1076 nmol/∗h).

#### 3.2.2. Associations with Diurnal and Awakening Cortisol in EO-CD

Pearson correlations between the predictors entered into a multiple linear regression model are shown in Table 2. Mainly, hyperactivity, impulsivity, and externalizing behavior were positively correlated, whereas CU traits were not related to any other variable. A multiple linear regression model with CU traits, impulsivity, hyperactivity, and externalizing behavior as independent predictors of AUC_*d*_ and AUC_*a*_, respectively, showed negative associations of CU traits with awakening cortisol (on a marginally significant level) and diurnal cortisol in participants with EO-CD (Table 3). These associations were observed in both the full and the final models. Hyperactivity was negatively associated with AUC_*a*_ but not with diurnal cortisol. Impulsivity was negatively associated with diurnal cortisol (AUC_*d*_) but not with cortisol after awakening (AUC_*a*_). Externalizing behavior was not associated with cortisol levels in either of the two models.

## 4. Discussion

The aim of this study was to investigate differences between a healthy control group and children and adolescents with EO-CD with regard to their diurnal and awakening cortisol levels and especially the impact of CU traits and comorbid ADHD symptomatology on cortisol levels. First, the data indicate largely comparable awakening and diurnal cortisol levels in participants with EO-CD compared to healthy controls. In EO-CD participants, however, cortisol levels showed a steeper decline between awakening and noon measurements. Second, CU traits were associated with both decreased diurnal and awakening cortisol levels, whereas externalizing behavior was not. Hyperactivity and impulsivity were negatively related to awakening and diurnal cortisol levels, respectively. Thus, when all variables were included in a multiple regression model, the findings indicated that CU traits in particular are related to reduced cortisol levels, while this was not the case with externalizing behavior in children with EO-CD. 

Cortisol levels throughout the day were numerically lower in EO-CD participants compared to healthy controls but not at a statistically significant level. This finding is in line with numerous other studies that reported no or small differences in group comparisons (meta-analysis by Alink and colleagues [8]). Regarding awakening cortisol levels, this study did not replicate a decreased CAR to be associated with antisocial behavior as reported previously in a methodologically very similar study by Popma and colleagues [10] or in a large study by Freitag and colleagues [50] investigating children with ADHD. Nevertheless, other investigations with a similar methodology, for example, that of Fairchild and colleagues [7], also did not report a reduced CAR. 

### 4.1. Influence of Callous-Unemotional Traits on Cortisol Levels

In this study, in participants with EO-CD, higher levels of CU traits were associated with significantly lower levels of cortisol throughout the day and—at a marginally significant level—in the first hour after awakening. The results are in line with findings from Loney and colleagues [15] in a study of a community sample of adolescents (ages from 12 to 18 years). The authors found that elevated CU traits were associated with low morning cortisol in boys, while subjects with antisocial behavior but no CU traits did not show reduced morning cortisol levels compared to healthy controls. This study expands upon that of Loney and colleagues [15] in that it assesses cortisol directly after awakening and repeatedly throughout the day. It is the first to report that diurnal cortisol levels and a reduced CAR are related to CU traits. A reduced CAR has been associated with smaller hippocampal volumes and changes in various regions of the limbic system [29] that in turn have been associated with psychopathy in the framework of reduced emotional learning [51]. Moreover, a diminished CAR has been interpreted to indicate a reduced reactivity of the HPA axis. The latter has been demonstrated in a study by Stadler and colleagues [27], who investigated boys with ADHD and found a blunted cortisol reactivity after induced stress in subjects with CU traits. The results in this study strengthen the hypothesis that specifically CU traits are associated with reduced cortisol levels more so than overall externalizing behavior. It is well known that antisocial behavior is of a very diverse etiology and that underlying biological mechanisms might vary largely. The data in this study point toward the necessity of further differentiating the diagnosis of a CD not only with regard to time of onset (i.e., early onset versus adolescent onset in the DSM-IV-TR) but also with regard to personality traits such as CU traits. This need has been played out in the proposal to add the CU specifier for CD in the upcoming DSM-5 [52]. 

### 4.2. Influence of ADHD Symptoms on Cortisol Levels

Furthermore, the results in this study indicate an independent negative relationship of ADHD related impulsivity and hyperactivity with diurnal and awakening cortisol, respectively. In assessing inattention, hyperactivity, and impulsivity on specific subscales in this study, the respective associations of specific ADHD related symptoms with cortisol were assessed independently for the first time. However, the results should be interpreted with caution. While previous studies have reported reduced cortisol levels in subgroups with predominantly hyperactive and impulsive behavior combined [12], no study thus far has assessed hyperactive symptoms independently. Moreover, most studies investigating the associations of ADHD related symptoms and cortisol report no associations with *basal* cortisol levels, whereas diminished cortisol levels in response to induced stress have been found repeatedly [14, 53]. One possible explanation for the observed associations between basal cortisol with ADHD related symptoms in this study might be that participants in the EO-CD group showed a more severe occurrence of hyperactivity and impulsivity than typically reported in epidemiological samples [54]. In line with our findings, Poustka and colleagues [28] found a negative association between poor impulse control and afternoon cortisol levels. However, impulsivity was measured as part of a psychopathy rating scale, which only partially overlaps with impulsiveness as a symptom of ADHD. 

### 4.3. Strengths and Limitations

The strengths of the study include the assessment of cortisol under well-controlled conditions with trained study nurses reliably assessing all cortisol samples in the inpatient unit and providing assistance with assessments at home. Furthermore, the sample was thoroughly characterized by employing elaborate up-to-date methodologies. In addition, the symptomatology of comorbid ADHD was measured on distinct subscales, differentiating the occurrence of inattention, hyperactivity, and impulsivity. The study also shows some limitations. First, for practical reasons, the cortisol assessments were performed on one day only; therefore, day-to-day variations could not be controlled for. Second, measures of daily activity that may have influenced cortisol levels and might have differed between groups were not assessed. A recent investigation however suggests that trait variables such as personality traits account for a large proportion of the variance in day-to-day variations of cortisol [55]. Third, the sample size is relatively small given small effect sizes of differences in cortisol profiles. Finally, the study group shows a relatively large age range including pre- and postpubertal adolescents. In the current analyses, however, pubertal status was not related to cortisol measures. 

## 5. Conclusion

In summary, this study did not identify reduced cortisol levels as a biological marker of antisocial behavior in EO-CD boys in general but does indicate a specific association between cortisol levels and CU traits. Furthermore, the results show reduced cortisol levels to be associated with poor impulse control, which is a trait prevalent in both ADHD and psychopathy. These findings suggest that hypoactivity of the HPA axis and thus a diminished responsivity to stress could be an important neurobiological underpinning of psychopathy in children and adolescents. Pending replication of our results, reduced cortisol levels could be considered as a mediator for the development of sustained antisocial behavior [56], which has been observed frequently in youths with psychopathic traits. Subsequently, these implications should be considered in the design of more individualized and targeted interventions.

## Figures and Tables

**Table 1:** Group comparisons.

	EO-CD		Healthy controls	
	Mean	SD	Mean	SD
Age (years)	11.9	2.4	12.5	2.7
IQ	97.0*	12.2	110.7*	14.8
Tanner-stage	1.9	1.6	2.3	1.7
Externalizing problems^a^	77.1*	6.8	49.3*	8.1
Internalizing problems^a^	71.2*	8.6	53.3*	9.5
Hyperactivity^b^	2.8*	2.6	0.2	0.5
Impulsivity^b^	2.4*	1.6	0.6	0.1
Callous-unemotional traits^a^	66.8*	9.5	48.4*	7.8
Awakening time (hours:min)	07:22	00:29	07:39	01:18
AUC: awakening cortisol (nmol/L∗h)	554.9	286.8	622.3	230.1
AUC: diurnal cortisol (nmol/L∗h)	2747.2	1643.8	3005.4	1769.0

Group comparisons by means of *t*-test; AUC: area under the curve; EO-CD: early-onset conduct disorder; min: minutes; ^a^ *T*-values are indicated; ^b^number of fulfilled DSM-IV criteria are indicated; *P < 0.01.

**Table 2:** Correlations between CU traits, impulsivity, hyperactivity, and externalizing behavior.

	1	2	3	4
CU traits	—			
Impulsivity	−0.33^‡^	—		
Hyperactivity	−0.12	0.60**	—	
Externalizing behavior	−0.06	0.40*	0.52**	—

**P < 0.01; *P < 0.05; ^‡^ *P* < 0.10.

**Table 3:** Associations of cortisol with callous-unemotional traits, ADHD-symptoms, and externalizing behavior.

	AUC_ a _				AUC_ d _			
	*B*	SE *B*	β	*P *	*B*	SE *B*	β	*P*
Full model								
Constant	2108.5	698.4		0.006	8673.0	2991.6		0.008
CU-traits	−8.9	5.2	−0.31	0.098	−49.6	22.2	−0.38	0.034
Externalizing behavior	−11.5	8.3	−0.27	0.179	−22.2	35.5	−0.12	0.536
Impulsivity	8.8	39.3	0.05	0.825	−67.3	105.4	−0.14	0.529
Hyperactivity	−32.3	24.6	−0.30	0.201	−349.5	168.3	−0.44	0.048
Final model								
Constant	1303.8	341.2		0.001	7211.4	1596.0		<0.001
CU-traits	−9.3	4.9	−0.32	0.068	−52.4	21.7	−0.40	0.023
Impulsivity					−459.5	130.1	−0.58	0.002
Hyperactivity	−44.4	18.1	−0.41	0.021				

Multiple linear regression; AUC_ a _: area under the curve of cortisol levels 1 h after awakening with respect to zero; AUC_ d _: area under the curve with respect to diurnal cortisol over 12 h; CU: callous-unemotional.

*R* ^2^ full model AUC_ a_ = 0.30;*R* ^2^ final model AUC_ a_ = 0.25; *R* ^2^ full model AUC_ d_ = 0.38; *R* ^2^ final model AUC_ d_ = 0.34.
